# Safety Assessment and Comparison of Sodium Selenite and Bioselenium Obtained from Yeast in Mice

**DOI:** 10.1155/2017/3980972

**Published:** 2017-10-30

**Authors:** Xinghua Wang, Yukun Yang, Hening Zhang, Ju Liu

**Affiliations:** ^1^School of Life Science, Shanxi University, Taiyuan 030006, China; ^2^Institute of Biotechnology, Shanxi University, Taiyuan 030006, China

## Abstract

Detailed safety assessment of sodium selenite and bioselenium (bio-Se) was conducted and the results were compared and discussed for the purpose of assessing safety of bio-Se for use in food applications. In this work, acute toxicity studies, micronucleus test, and sperm aberration study in mice, 30-day feeding test of mice, were conducted to evaluate the toxicity of bio-Se obtained from yeast with different fermentation time (transformative time: one month, three months, and six months), and the results were compared with that of inorganic Se (sodium selenite). LD_50_ of sodium selenite was calculated to be 21.17 mg/kg. LD_50_ of bio-Se obtained from yeast with different fermentation time was calculated to be 740.2 mg/kg, 915.3 mg/kg, and 1179.0 mg/kg, respectively. In the genotoxicity test, bio-Se did not show cytotoxicity and genotoxicity of mice while sodium selenite at all dose groups was significantly different from the negative group. In the 30-day subchronic oral toxicity study, sodium selenite may slow down the growth of the mice and lead to organic damage to some extent. Bio-Se had facilitated effect towards the body weight of the mice and had no significant effect on the shape and function of the important organs of the mice.

## 1. Introduction

Selenium (Se) is a naturally occurring metalloid element, which is essential to the health of human and other animals in trace amounts but is harmful in excess. As a micronutrient, Se owns multiple biological functions including regulation of vitamins absorption, participation in electron transfer, body metabolism regulation, affection towards the reproductive function of humans and animals, antitumor, free radical scavenger, antiaging, and antagonism of toxic elements, playing important roles in improvement of the human body's immune system and the prevention of various diseases [1–4]. Currently, many Se-enriched foods are available in the market, in which Se additives are mainly in the form of inorganic and organic Se. Generally, Se in organic forms has better bioavailability and is less toxic compared with inorganic selenium with poor conversion and high toxicity to some extent [5–8]. Nowadays, various available sources of organic Se, namely, Se-enriched yeast [9–11], selenomethionine [12–14], Se-enriched probiotics [15], heterotrophically produced Se-enriched alga [16, 17], and Se-enriched plants [18, 19], have been investigated over time. Among these organic Se, the main organic Se source used in practice is Se-enriched yeast, in which Se is present predominantly (94 ± 5%) in the form of protein-bound L-selenomethionine [20]. As a kind of organic Se, Se-enriched yeast owned the advantages of high effectiveness, lower toxicity, and better nutrition. However, the composition of the raw products of Se-enriched yeast (obtained by culture and whizzer) is complex and may contain unconverted inorganic selenium, possessing obstacles for the practice application in food industry.

Therefore, in this work, further works were made for the more secure and easily adsorbed bio-Se. The yeast cells were lysed by sonication after a period of growth with sodium selenite and dialyzed to remove the free inorganic selenium. Finally, the bio-Se obtained from yeast was produced. Theoretically, compared with raw products of Se-enriched yeast, bio-Se is a safe and effective formulation which holds important practical value. However, the toxicology of the bio-Se obtained from yeast was not fully convinced yet.

Because external intake is the most important source of Se in humans, understanding the toxicity of sodium selenite and bio-Se obtained from yeast is the key to the assessment of Se related health risks. In this work, acute toxicity studies, micronucleus test, and sperm aberration study in mice, 30-day subchronic oral toxicity study of mice, were conducted to evaluate the toxicity of bio-Se obtained from yeast with different fermentation time (transformative time: one month, three months, and six months), and the results were compared with that of inorganic Se (sodium selenite). The purpose of this work is to summarize the results obtained from acute toxicity, genetic toxicity, and subchronic toxicity of bio-Se obtained from yeast in mice and compared with the safety assessment of sodium selenite, as well as providing theoretical basis for the application of bio-Se obtained from yeast.

## 2. Materials and Methods

### 2.1. Chemicals and Instruments

Fetal bovine serum (FBS) and Giemsa dye were obtained from Seajet Scientific (Beijing, China) and Zhejiang Tianhang Biotechnology Co., Ltd. (Hangzhou, China), respectively. Other chemicals used in the experiments were purchased from Tianjin Chemical Reagent Research Institute. Se standard solution (1000 *μ*g/mL) was obtained from China Iron and Steel Research Technology Group Co., Ltd. (Beijing, China).

Flame Atomic Absorption Spectrophotometer (FAAS, A6300, Japan Shimadzu Hong Kong Co., Ltd.) equipped with LH-2A Hydride Generator (Brother Chuanghekemao Co., Ltd., Beijing, China) was used for the determination of Se content. JY92-II ultrasonic cell grinder was purchased from Xinzhike Institute (Ningbo, China). Dialysis bag MD34 (8000–14000) was obtained from Sdarbio Life Science Company.

### 2.2. Preparation Methods of Bio-Se Obtained from Yeast

Bio-Se obtained from yeast was prepared and purified by our laboratory. Cell culture is as follows: yeast strain:* Saccharomyces cerevisiae *D254; fermentation medium: 2% glucose, 1% peptone, 0.5% yeast extract, 0.1% KH_2_PO_4_, 0.05% MgSO_4_·7H_2_O, 0.01% CaCl_2_·6H_2_O, and 0.5% (NH_4_)_2_SO_4_. The optimal conditions for Se accumulation in yeast were optimized using response surface method and were found to be a temperature of 28.6°C, pH value of 6.3, and an added concentration of Na_2_SeO_3_ of 81.3 mg/L, which resulted in the maximum Se accumulation. Under these conditions, the yield of bio-Se was predicted to be 1544.165 *μ*g/L (using the amount of Se to calculate).

After the yeast cells grew to the required quantity, samples were collected and sonicated, and the lysed cell contents were dialyzed in dialysis bags (MD34) against distilled water with 1 ml of cysteine solution (stock concentration of 150 g/L). During dialysis, the water was consistently changed until the solution did not fade within one minute after the addition of methylene blue, indicating that all the inorganic selenium was eliminated. The selenium incorporation in the extract was measured using FAAS after the extract was digested with mixed acid (HNO_3_ : HClO_4_ = 4 : 1).

### 2.3. Animals

Male and female Kunming species of mice were obtained from Experimental Animal Center of Shanxi Medical University (Taiyuan, China). The mice were fasted for 8 h and with free access to water prior to the experiments. Throughout the experiments, animals were processed according to the suggested ethical guidelines for the care of laboratory animals.

### 2.4. Acute Toxicity Study of Sodium Selenite and Bio-Se in Mice

The acute toxicity test of sodium selenite and bio-Se in mice was conducted and the results were compared.

60 healthy Kunming mice (6–8-week-old mice, half male and half female) at average body weight (bw) of 18–20 g were allotted to six groups (each group of ten, half male and half female). The oral dose of sodium selenite experimental group was 5.56, 8.33, 12.50, 18.75, 28.12, and 42.19 mg/kg bw. After oral administration, the daily behavior, group behavior, toxicity, and death of the mice were observed and recorded for two weeks.

As for bio-Se with different fermentation time, 120 healthy Kunming mice (6–8-week-old mice, half male and half female) at average body weight of 18–20 g were allotted to twelve groups (each group of ten, half male and half female). Acute toxicity of bio-Se with fermentation time of one month, three months, and six months was measured using the oral dose of 5240, 7860, 11790, and 17685 mg/kg bw. After oral administration, the daily behavior, group behavior, toxicity, and death of the mice were observed and recorded.

### 2.5. Genetic Toxicity Test of Sodium Selenite and Bio-Se in Mice

Bone marrow micronucleus test and sperm aberration experiment were used to evaluate the genetic toxicity of sodium selenite and bio-Se.

#### 2.5.1. Bone Marrow Micronucleus Test

The experiments were carried out according to Von Ledebur and Schmid [21]. 80 healthy Kunming mice (6–8-week-old mice, half male and half female) at average body weight of 18–20 g were allotted to eight groups. 1/2LD_50_, 1/4LD_50_, and 1/8LD_50_ (i.e., 10.59, 5.29, and 2.65 mg/kg bw) of sodium selenite were used as high, middle, and low dose group. Bio-Se with fermentation time of six months was used for evaluation. 1/2LD_50_, 1/4LD_50_, and 1/8LD_50_ (i.e., 589, 2950, 1470 mg/kg bw) were used as high, middle, low dose group. A positive control group (cyclophosphamide, 40 mg/kg bw) and a negative control group (distilled water) were included.

Sodium selenite or bio-Se was orally administered within 30 h; that is, the interval between the first and the second test was 24 h. After 6 h of the second test, the tested mice were euthanized by cervical dislocation, and their bone marrow cells were flushed from both femurs into fetal calf serum. After centrifugation, the bone marrow cells were smeared on glass slides, coded for blind analysis, air-dried, and fixed with absolute methanol for 5 min at room temperature. The smears were stained with Giemsa, dibasic sodium phosphate, and monobasic sodium phosphate to detect micronucleated polychromatic erythrocytes (MNPCE). For each animal, 1000 polychromatic erythrocytes (PCE) were counted to determine the frequency of MNPCE using light microscopy. Genetic toxicity and antigenotoxicity were assessed by the frequency of MNPCE, whereas cytotoxicity and anticytotoxicity were evaluated by the PCE and normochromatic erythrocytes (NCE) ratio (PCE/NCE).

To analyze the genotoxic and antigenotoxic activities of sodium selenite or bio-Se using the mouse bone marrow micronucleus test, the frequency of MNPCE in the treated groups was compared to the results obtained from the negative control group using SPSS17.0 software for significant differences analysis (*P* ≥ 0.05 indicated no significant difference, *P* < 0.05 considered a statistically significant difference).

#### 2.5.2. Sperm Aberration Test

For the sperm aberration test, 80 Kunming male mice were used. Animal grouping and the administered dosages were the same as those in the bone marrow micronucleus test. The mice were intragastrically administered various dosages daily for 5 days and were euthanized by cervical dislocation 35 days after the first administration. The bilateral epididymis was collected, sectioned and stained by routine clinical protocol, and observed under the microscope.

A thousand sperm were observed for each mouse to record the numbers of aberrated sperm, and aberration ratios were analyzed and calculated. Significant differences were analyzed with SPSS software (*P* ≥ 0.05 indicated no significant difference, *P* < 0.05 considered a statistically significant difference).

### 2.6. 30-Day Subchronic Oral Toxicity Test

160 healthy Kunming mice (6 weeks old, half male and half female) at average body weight (bw) of about 16–20  g were allotted to eight groups (each containing 10 females and 10 males). The highest dose group was set at 20% of LD_50_. Three dose groups of sodium selenite were set at 1.41 mg/kg, 2.12 mg/kg, and 3.16 mg/kg, and three dose groups of bio-Se were set at 786.3 mg/kg, 1179.5 mg/kg, and 1769.3 mg/kg. A control group formed by 20 mice consumed distilled water during a 30-day period.

The daily action, the occurrence of the toxicity, and the death of the mice were observed and recorded daily after the start of the test. At the end of the test, fasting for 12 h overnight, the mice were euthanized by cervical dislocation. The weights of mice were recorded once a week. The weights of heart, liver, spleen, and kidney were recorded for the calculation of the organ coefficient. SPSS17.0 software was used for significant difference analysis (*P* ≥ 0.05 indicated that there was no significant difference, *P* < 0.05 considered a statistically significant difference).

## 3. Results and Discussion

### 3.1. The Acute Toxicity Assay of Sodium Selenite and Bio-Se

To assess the safety of different selenium source (sodium selenite, bio-Se obtained from yeast with different fermentation time (1 month, 3 months, 6 months)), LD_50_ test was first conducted. After administration of different Se sources, it was found that male and female mice with three dose levels of sodium selenite (12.50 mg/kg bw, 28.13 mg/kg bw, 42.19 mg/kg bw) showed convulsion and piloerection and were gathered into piles with varying degrees. Small part of mice showed the symptoms of diarrhea, and all the mice died within one week. At the dose groups of bio-Se with different fermentation time, the tested mice appeared healthy with normal appearance and little mice showed the above phenomenon. The results of acute toxicity test of sodium selenite were shown in Table 1.

Thus, LD_50_ of sodium selenite was calculated to be 21.17 mg/kg. According to the classification standard of acute toxicity, sodium selenite belongs to the category 5 (highly toxicity). Meanwhile, the acute toxicity test results of bio-Se obtained from yeast were shown in Table 2.

Thus, LD_50_ of bio-Se obtained from yeast with different fermentation time (1 month, 3 months, 6 months) was calculated to be 740.2 mg/kg, 915.3 mg/kg, and 1179 mg/kg, respectively. According to the classification standard of acute toxicity, bio-Se obtained from yeast with different fermentation time belongs to category 2 (the actual nontoxicity).

LD_50_ of bio-Se obtained from yeast with different fermentation time was dramatically decreased compared with that of sodium selenite. LD_50_ of bio-Se obtained from yeast with fermentation time of 6 months even reached 1179 mg/kg. In other words, the toxicity of bio-Se was greatly reduced through biotransformation compared with inorganic selenium. The toxicity of bio-Se could be further reduced as the fermentation time was prolonged. According to the food toxicology measurement index, LD_50_ should be greater than 10 times the dose which the people may normally be exposed to and then genotoxicity test could be carried next. While bio-Se is considered as selenium supplement, the Se intake of 50 micrograms per day is recommended in China, which was much less than LD_50_ of bio-Se. So, the next stage of mutagenicity test could be carried out.

### 3.2. Genetic Toxicity Test of Sodium Selenite and Bio-Se

#### 3.2.1. Bone Marrow Micronucleus Test of Sodium Selenite and Bio-Se

Tables 3 and 4 showed the results of cytotoxic and genotoxic activities in the bone marrow of mice treated with sodium selenite and bio-Se based on micronucleus rate and polychromatic erythrocyte/normochromatic erythrocyte (PCE/NCE) ratio. As expected, the negative control group (distilled water) exhibited low micronucleus rate level, whereas the positive control group of micronucleus rate was significantly higher than the negative control group (*P* < 0.05), indicating that the experimental system is sensitive to the mutation.

According to Table 3, the groups which received 1/8LD_50_, 1/4LD_50_, and 1/2LD_50_ amount of sodium selenite exhibited mean micronucleus rate of 9.3, 9.5, and 10.3‰, respectively (the negative control group exhibited mean micronucleus rate of 3.2‰). At all tested doses, sodium selenite caused significant increase in the micronucleus rate compared to the negative control group (*P* < 0.05). Meanwhile, PCE/NCE ratio of sodium selenite was 0.54, 0.43, and 0.16, respectively, which were significantly different compared with the negative control group, and its value was not in the normal range (0.6~1.2 belong to the normal range, <0.6 represents that formation of PCE was inhibited). In the bone marrow micronucleus test, sodium selenite could increase the micronucleus rate of bone marrow cells in mice, indicating that sodium selenite had mutagenicity.

According to Table 4, the micronucleus rates of the three dose groups of bio-Se were not significantly different from that of the negative control group, while the result of PCE/NCE ratio in the three dose groups of bio-Se was not significantly different from that of the negative control group (*P* > 0.05).

In the experiment, the ratio of PCE/NCE of bio-Se groups was in the normal range, proving bio-Se does not have the role of cell chromosome mutation. From the experimental results of bone marrow micronucleus test, it could be concluded that the mutagenicity of bio-Se obtained from yeast on mice decreased significantly compared with sodium selenite groups.

#### 3.2.2. Sperm Aberration Test of Sodium Selenite and Bio-Se

From Table 5, the sperm aberration rate of the positive control group was significantly higher than that of the negative control group (*P* < 0.05), indicating that the experimental system was sensitive to the mutagenicity. Sperm aberration rates in three dose groups of sodium selenite were significantly different from that of the negative control group (*P* < 0.05), indicating that sodium selenite could induce mouse sperm more prone to distortion and thus lead to the rising of sperm aberration rate.

From Table 6, the sperm aberration rate of the positive control group was significantly higher than that of the negative control group (*P* < 0.05), indicating that the experimental system was sensitive to the mutagenicity. Sperm aberration rate in the three groups of bio-Se has no significant difference compared with the negative control group (*P* > 0.05), indicating that bio-Se could not cause sperm squirrel distortion of mice.

Therefore, the results indicated that sodium selenite existed genetic toxicity, while bio-Se did not cause mutagenic effects on mouse cells.

### 3.3. Subchronic Toxicity Test of Sodium Selenite and Bio-Se

The 30-day subchronic toxicity test was conducted after 30 days of continuous administration of the test animals for a more profound understanding of the toxicological properties of sodium selenite and bio-Se.

#### 3.3.1. General Behavior Observation

During the experiment, it was observed that the mice in the high dose group of sodium selenite had a decrease in activity compared with the negative control group. There was no significant general behavioral difference observed between control group and other groups (middle, low dose group of sodium selenite, and three groups of bio-Se).

#### 3.3.2. Effects of Sodium Selenite and Bio-Se on Body Weight of Mice

The effect of sodium selenite and the bio-Se groups on body weight of mice was recorded and the results were shown in Tables 7 and 8.

As shown in the Table 7 the weekly body weight gains of mice in sodium selenite groups were lower than that of the negative group starting from the second week, indicating that sodium selenite may have inhibition effect on the mice growth. According to the data analysis, there was significant difference between the three dose groups and the negative control group at the third week, and there was highly significant difference between the high dose group and the negative control group at the fourth week. It should be noted that there was a significant difference between the low dose group and the negative control group at the second week. While other groups did not show significant difference, the probable reason was that effect of Se with different concentrations on the growth was different. This means that only the concentration of the test substance at a certain range could play an obvious effect on the results.

As shown in Table 8, the weight of the mice in the high, middle, and low groups of bio-Se during the 30-day test period was increasing continuously. Compared with the negative control group, there was no significant difference in the weight of low and middle dose group, while in the high dose group at third and fourth week, there was significant difference in the weight gain compared with control group.

Through the experimental results of weight changes, it could be concluded that bio-Se had weight gain effect on the growth of the mice compared with the inhibition effect on the mice growth of sodium selenite. There was no significant negative effect, and the body weight of the mice in the high dose group of bio-Se was significantly increased compared with the negative control group.

#### 3.3.3. Effects of 30-Day Subchronic Toxicity Test on Organ Coefficient of Mice Using Sodium Selenite and Bio-Se

The organ coefficient is a good way to express the toxicological properties of the test substance towards organ, which could explain that the pathology of the tissue has changed. When the organ coefficient decreases, this decrease indicates the atrophy and degeneration of the organ, while the increment of organ coefficient indicates that the organ had hypertrophy or other lesions.

Effects of 30-day subchronic toxicity test on organ coefficient (organ-to-body weight ratio) of mice using sodium selenite and bio-Se were shown in Tables 9 and 10. According to the data from Table 9, the organ coefficients of heart, spleen, and kidney in high dose group had highly significant difference (*P* < 0.01) compared with control group. In the low and middle dose groups, there was significant difference or highly significant difference in different organ coefficients compared with the control group. The liver and kidney were heavier after the intake of excess sodium selenite, and the synthesis function of liver albumin decreased or albumin precipitated between cells and cells, resulting in decreased plasma osmotic pressure, and thus liver and kidney cells were swollen. Spleen coefficient was lower than the negative control group, indicating that the spleen had a small degree of atrophy. The reason may be that sodium selenite had inhibited the growth of spleen. It was observed that there was damage of heart, liver, spleen, and kidney after the anatomy.

The effect of 30-day subchronic toxicity test using bio-Se on the organ coefficient of the mice was shown in Table 10. The organ coefficient of heart, liver, spleen, and kidney in three doses groups had no statistical difference (*P* > 0.05) when compared with the control group. There was no abnormal change in the main organs after the anatomy of mice. Therefore, it could be concluded that the bio-Se has no significant effect on the shape and function of the organs of the important organs of the mice. After fermenting process of yeast, the toxicity of bio-Se was significantly lower than that of sodium selenite, which laid the foundation for subsequent food application.

## 4. Conclusion

Detailed safety assessment of sodium selenite and bio-Se was conducted and the results were compared and discussed in this work. In conclusion, in the acute toxicity test, LD_50_ of sodium selenite was calculated to be 21.17 mg/kg. LD_50_ of bio-Se obtained from yeast with different fermentation time (1 month, 3 months, 6 months) was calculated to be 0.7402 g/kg, 0.9153 g/kg, and 1.179 g/kg, respectively. In the genotoxicity test, micronucleus rate (‰), PCE/NCE, and sperm aberration rate (%) of sodium selenite at all dose groups were significantly different from the negative group, while bio-Se did not have significant difference with the negative group. In the 30-day subchronic oral toxicity study, sodium selenite may slow down the growth of rats and lead to organic damage to some extent. Bio-Se had facilitated effect towards the body weight of the mice and had no significant effect on the shape and function of the important organs of the mice. Therefore, from the results of the study presented herein, it may be concluded that bio-Se obtained from yeast after fermentation and transformation of sodium selenite had no significant toxicity and could be considered safe.

## Figures and Tables

**Table 1:** Statistical results of acute toxicity test for sodium selenite.

Category (mg/kg bw)	Number of tested animals	Number of dead animals	Number of surviving animals	Mortality (%)
5.55	10	0	10	0
8.33	10	1	9	10
12.50	10	3	7	30
28.13	10	8	2	80
42.19	10	10	0	100

**Table 2:** Statistical results of mortality in acute toxicity test for bio-Se obtained from yeast with different fermentation time.

Category	Mortality (%)			
	5240 mg/kg bw	7860 mg/kg bw	11790 mg/kg bw	17685 mg/kg bw
1	20	40	100	100
2	10	20	80	100
3	0	20	30	100

*Note*. 1, 2, and 3 represent the bio-Se obtained from yeast with different fermentation time (1 month, 3 months, 6 months), respectively.

**Table 3:** The experimental results of sodium selenite in bone marrow micronucleus.

Category	Dose (mg/kg)	Number of tested animals	Check PCE number	MNPCE number	Micronucleus rate (‰)	PCE/NCE
1/8LD_50_	2.65	10	10 × 1000	93	9.30 ± 0.423^*∗*^	0.54 ± 0.25^*∗*^
1/4LD_50_	5.29	10	10 × 1000	95	9.50 ± 0.696^*∗*^	0.43 ± 0.37^*∗*^
1/2LD_50_	10.59	10	10 × 1000	103	10.3 ± 0.537^*∗*^	0.16 ± 0.21^*∗*^
Negative control group	Distilled water	10	10 × 1000	32	3.20 ± 0.446	1.00 ± 0.38
Positive control group	Cyclophosphamide	10	10 × 1000	124	12.40 ± 1.511^*∗*^	0.70 ± 0.12^*∗*^

*Note*. *∗* indicated that there was a significant difference compared with the negative control group (*P* < 0.05).

**Table 4:** The experimental results of bio-Se in bone marrow micronucleus.

Category	Dose (g/kg)	Number of tested animals	Check PCE number	MNPCE number	Micronucleus rate (‰)	PCE/NCE
1/8LD_50_	1.47	10	10 × 1000	28	2.80 ± 0.493	1.08 ± 0.34
1/4LD_50_	2.95	10	10 × 1000	34	3.40 ± 0.424	1.09 ± 0.41
1/2LD_50_	5.89	10	10 × 1000	32	3.20 ± 0.437	1.00 ± 0.60
Negative control group	Distilled water	10	10 × 1000	32	3.20 ± 0.557	0.90 ± 0.29
Positive control group	Cyclophosphamide	10	10 × 1000	124	12.40 ± 1.459^*∗*^	0.74 ± 0.17^*∗*^

*Note*. *∗* indicates that there were significant differences with the negative control group (*P* < 0.05).

**Table 5:** The experimental results of sodium selenite in sperm aberration test.

Category	Dose (mg/kg)	Number of tested animals	Sperm count	Distorted sperm count	Sperm aberration rate (%)
1/2LD_50_	10.59	10	10 × 1000	395	3.95 ± 0.443^*∗*^
1/4LD_50_	5.29	10	10 × 1000	404	4.04 ± 0.428^*∗*^
1/2LD_50_	2.65	10	10 × 1000	420	4.20 ± 0.561^*∗*^
Negative control group	Distilled water	10	10 × 1000	269	2.69 ± 0.658
Positive control group	Cyclophosphamide (40 mg/kg)	10	10 × 1000	445	4.45 ± 1.516^*∗*^

*Note*. *∗* indicates that there is a significant difference compared with the negative control group (*P* < 0.05).

**Table 6:** The experimental results of bio-Se in sperm aberration test.

Category	Dose (mg/kg)	Number of tested animals	Sperm count	Distortion sperm count	Sperm aberration rate (%)
1/2LD_50_	1.47	10	10 × 1000	243	2.43 ± 0.532
1/4LD_50_	2.95	10	10 × 1000	283	2.83 ± 0.547
1/2LD_50_	5.89	10	10 × 1000	252	2.52 ± 0.475
Negative control group	Distilled water	10	10 × 1000	269	2.69 ± 0.658
Positive control group	Cyclophosphamide (40 mg/kg)	10	10 × 1000	445	4.45 ± 1.158^*∗*^

*Note*. *∗* indicates that there are significant differences (*P* < 0.05) with negative control group.

**Table 7:** Effects of sodium selenite on body weight using 30-day feeding experiment.

Group	Average weight (g)				
	Prior to the experiment	1 week (mean ± SD)	2 weeks (mean ± SD)	3 weeks (mean ± SD)	4 weeks (mean ± SD)
Negative control	16.51 ± 0.97^A^	22.53 ± 4.47^A^	26.53 ± 3.24^A^	29.41 ± 3.34^A^	35.42 ± 2.67^A^
Low dose group	16.82 ± 1.42^A^	23.71 ± 2.06^A^	23.36 ± 1.69^a^	23.32 ± 1.69^a^	22.71 ± 2.66^B^
Middle dose group	16.81 ± 1.52^A^	22.43 ± 2.60^A^	24.74 ± 2.34^A^	21.33 ± 2.49^b^	24.81 ± 2.31^b^
High dose group	17.01 ± 1.45^A^	24.30 ± 2.98^A^	24.52 ± 2.78^A^	23.40 ± 2.41^a^	23.11 ± 2.84^B^

*Note*. ^a,b,c,d,A,B^Letters in common in the same column do not present significant difference (*P* > 0.05), for example, A and A, b and b. Same letters in uppercase and lowercase or different letters in lowercase in the same column present significant difference (*P* < 0.05), for example, B and b, a and c. Different letters in uppercase or different letter in uppercase and lowercase present extremely significant difference, for example, A and B, A and b.

**Table 8:** Effects of bio-Se on body weight using 30-day feeding experiment.

Group	Average weight (g)				
	Prior to the experiment	1 week (mean ± SD)	2 weeks (mean ± SD)	3 weeks (mean ± SD)	4 weeks (mean ± SD)
Negative control	19.96 ± 1.46^A^	24.72 ± 2.29^A^	26.86 ± 2.37^A^	28.71 ± 2.66^A^	29.86 ± 2.52^A^
Low dose group	19.77 ± 1.77^A^	25.01 ± 2.68^A^	27.30 ± 3.27^A^	29.50 ± 3.32^A^	30.43 ± 3.37^A^
Middle dose group	19.85 ± 1.77^A^	25.78 ± 2.97^A^	28.77 ± 3.21^A^	30.75 ± 3.06^A^	31.78 ± 3.10^A^
High dose group	20.25 ± 1.63^A^	25.32 ± 2.89^A^	28.04 ± 3.27^A^	31.16 ± 3.24^B^	33.65 ± 3.40^B^

*Note*. ^A  B^Letters in common in the same column do not present significant difference (*P* > 0.05), for example, A and A, B and B. Different letters in the same column present significant difference (*P* < 0.05), for example, A and B.

**Table 9:** Effects of 30-day subchronic toxicity test on organ coefficient of mice using sodium selenite.

Group	Heart/bw% (mean ± SD)	Liver/bw% (mean ± SD)	Spleen/bw% (mean ± SD)	Kidney/bw% (mean ± SD)
Negative control	0.35 ± 0.05^b^	4.36 ± 0.38^b^	0.23 ± 0.03^a^	0.68 ± 0.05^c^
Low dose group	0.36 ± 0.03^b^	4.70 ± 0.38^b^	0.22 ± 0.02^B^	0.71 ± 0.06^b^
Middle dose group	0.39 ± 0.04^a^	5.03 ± 0.62^A^	0.19 ± 0.01^a^	0.74 ± 0.04^B^
High dose group	0.43 ± 0.06^A^	5.41 ± 0.78^b^	0.16 ± 0.01^B^	0.79 ± 0.07^A^

*Note*. ^a,b,c,A,B^Letters in common in the same column do not present significant difference (*P* > 0.05), for example, A and A, b and b. Same letters in upper and lower case or different letters in lower-case in the same column present significant difference (*P* < 0.05), for example, B and b, b and c. Different letters in uppercase or different letters in uppercase and lowercase present extremely significant difference (*P* < 0.01), for example, A and B, B and c.

**Table 10:** Effects of 30-day feeding test on organ coefficient of mice using bio-Se.

Group	Heart/bw% (mean ± SD)	Liver/bw% (mean ± SD)	Spleen/bw% (mean ± SD)	Kidney/bw% (mean ± SD)
Negative control	0.53 ± 0.04	4.78 ± 0.33	0.37 ± 0.04	1.39 ± 0.12
Low dose group	0.54 ± 0.04	4.73 ± 0.25	0.38 ± 0.04	1.42 ± 0.14
Middle dose group	0.51 ± 0.03	4.95 ± 0.40	0.35 ± 0.04	1.38 ± 0.13
High dose group	0.49 ± 0.05	4.63 ± 0.39	0.36 ± 0.06	1.39 ± 0.14
